# Supplementary figures and images for: HPV-Associated Sexually Transmitted Infections in Cervical Cancer Screening: A Prospective Cohort Study
Source: Viruses. 2025 Feb 11;17(2):247. doi: 10.3390/v17020247 (PMC11861167; doi:10.3390/v17020247)

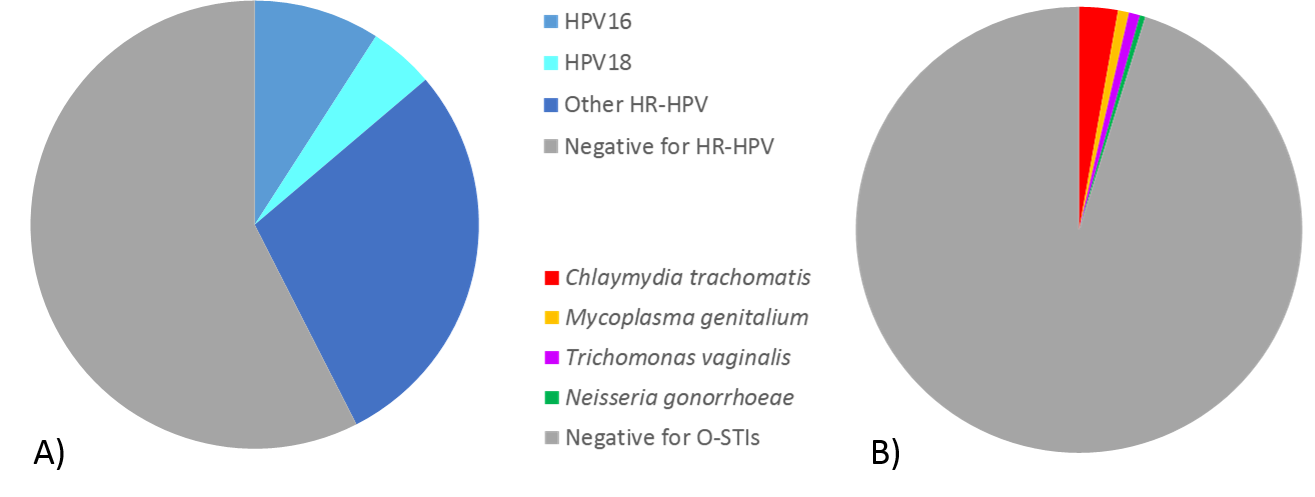

Supplement: Supplementary file 1 [file viruses-17-00247-s001.zip › SF1.png]

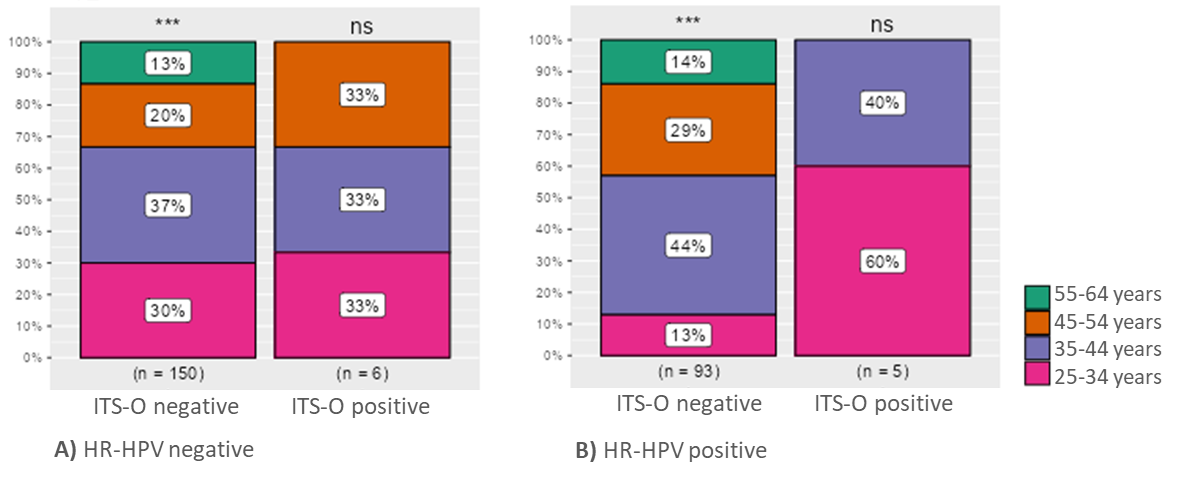

Supplement: Supplementary file 1 [file viruses-17-00247-s001.zip › SF2.png]

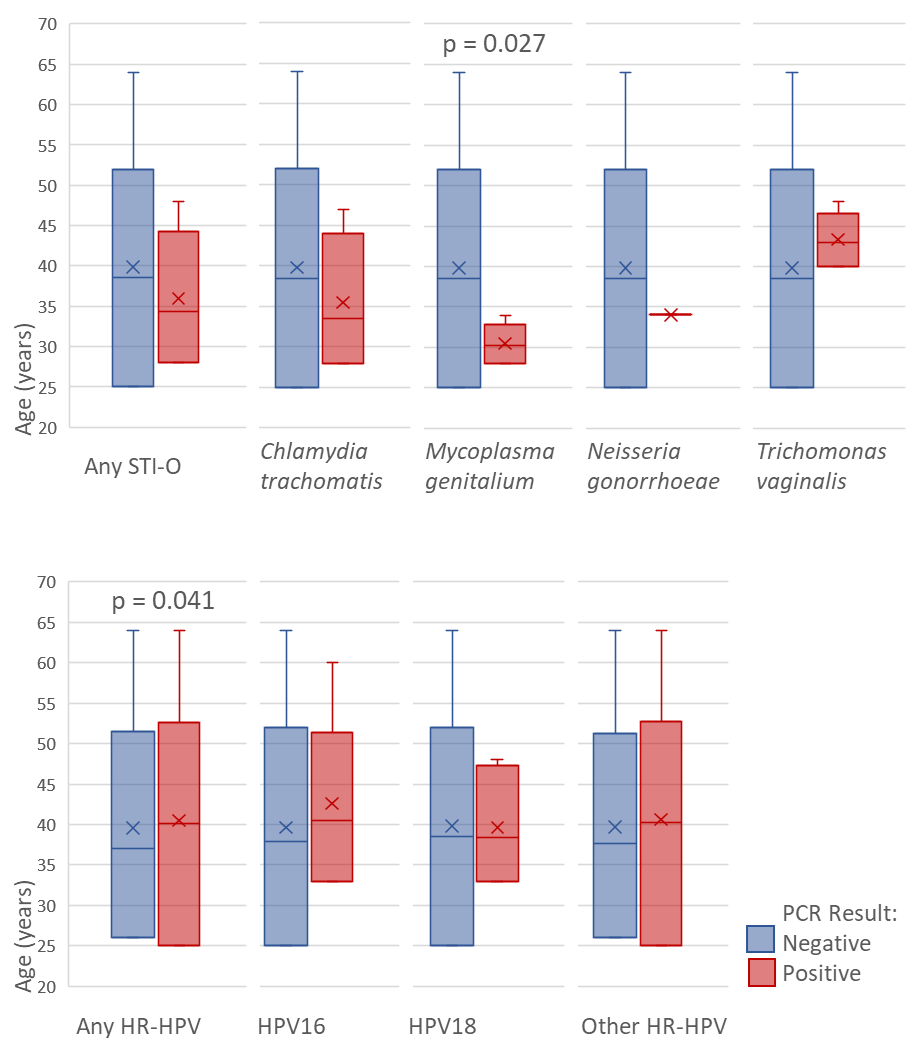

Supplement: Supplementary file 1 [file viruses-17-00247-s001.zip › SF3.png]
